# Evaluating the Cisplatin Dose Dependence of Testicular Dysfunction Using Creatine Chemical Exchange Saturation Transfer Imaging

**DOI:** 10.3390/diagnostics12051046

**Published:** 2022-04-21

**Authors:** Reika Sawaya, Sohei Kuribayashi, Junpei Ueda, Shigeyoshi Saito

**Affiliations:** 1 Department of Medical Physics and Engineering, Area of Medical Imaging Technology and Science, Division of Health Sciences, Osaka University Graduate School of Medicine, Suita 565-0871, Osaka, Japan; u010443b@ecs.osaka-u.ac.jp (R.S.); uedaj@sahs.med.osaka-u.ac.jp (J.U.); 2 Department of Medical Technology, Osaka University Hospital, Suita 565-0871, Osaka, Japan; 3 Department of Urology, Osaka University Graduate School of Medicine, Suita 565-0871, Osaka, Japan; kuribayashi@uro.med.osaka-u.ac.jp; 4 Department of Advanced Medical Technologies, National Cerebral and Cardiovascular Center Research Institute, Suita 564-8565, Osaka, Japan

**Keywords:** chemical exchange saturation transfer, CEST, radiology, animal study, creatine, cisplatin-treated model

## Abstract

Chemical exchange saturation transfer (CEST) imaging is a non-invasive molecular imaging technique for indirectly measuring low-concentration endogenous metabolites. Conventional CEST has low specificity, owing to the effects of spillover, magnetization transfer (MT), and T_1_ relaxation, thus necessitating an inverse Z-spectrum analysis. We aimed to investigate the usefulness of inverse Z-spectrum analysis in creatine (Cr)-CEST in mice, by conducting preclinical 7T-magnetic resonance imaging (MRI) and comparing the conventional analysis metric magnetization transfer ratio (*MTR_conv_*) with the novel metric apparent exchange-dependent relaxation (*AREX*). We performed Cr-CEST imaging using 7T-MRI on mouse testes, using C57BL/6 mice as the control and a cisplatin-treated model. We prepared different doses of cisplatin to observe its dose dependence effect on testicular function. CEST imaging was obtained using an MT pulse with varying saturation frequencies, ranging from −4.8 ppm to +4.8 ppm. The application of control mouse testes improved the specificity of the CEST effect and image contrast between the testes and testicular epithelium. The cisplatin-treated model revealed impaired testicular function, and the Cr-CEST imaging displayed decreased Cr levels in the testes. There was a significant difference between the low- and high-dose models. The MTR values of Cr-CEST reflected the cisplatin dose dependence of testicular dysfunction.

## 1. Introduction

Chemical exchange saturation transfer (CEST) imaging is a non-invasive molecular imaging technique that indirectly measures low-concentration endogenous metabolites [1]. In CEST imaging, a magnetization transfer (MT) pulse selectively saturates protons, following which this magnetic saturation is transferred between the excited metabolite protons and non-excited water protons through chemical exchange. This decreases the water signal. The measurement of this attenuation helps to indirectly determine the number of low-concentration solutes. Exchangeable protons include amino protons (-NH_2_), amide protons (-NH), and hydroxyl protons (-OH) [1]. The chemical exchange rate depends on the pH level, metabolite concentration, and temperature [2]. Furthermore, the CEST signal is affected by various physical factors, such as the radiofrequency (RF) magnitude, magnetic field strength, and B_0_ and B_1_ homogeneity [3,4,5]. In conventional CEST imaging of endogenous metabolites in vivo, the Z-spectrum not only reflects the CEST signal, but also the semi-solid macromolecular MT effect, the direct water proton saturation (spillover) effect, and the T_1_ relaxation of the water protons [6,7]. Therefore, the specificity of the CEST signal can be compromised by its effects.

Reliable CEST quantification warrants the elimination of factors that affect the signal. Researchers have used several approaches, such as the three-point method [8], chemical exchange rotation transfer [9], Lorentzian-line-fit analysis, and Lorentzian difference model fitting [10], to solve these problems. However, these methods generate insufficient correction. For more accurate detection of CEST effects, Zaiss et al. proposed a novel evaluation method that isolates and corrects the effects of spillover, MT, and T_1_ relaxation on the CEST signal, using the inverse metric of the Z-spectrum (1/Z) [6]. They presented a novel index, the *MTRrex*, which eliminates spillover and MT effects. Moreover, the index that compensates for T_1_ relaxation is termed the apparent exchange-dependent relaxation (*AREX*) [5,11].

Previous researchers have performed CEST imaging on various metabolites, such as amide protons [4], glutamate (Glu) [12], lactate [13], creatine (Cr) [14], and glycosaminoglycans (GAG) [15], in numerous organs. In particular, Cr-CEST imaging is useful for detecting Cr in the skeletal muscles [16,17] and brain [13,18]. Recently, researchers have reported on the usefulness of Cr-CEST imaging in the testis when examining Cr metabolites that have been altered by testicular torsion and radiation damage [19,20]. However, they performed Cr-CEST imaging using the conventional method, such that the specificity of the CEST signal could be compromised by spillover, the MT effect, and T_1_ relaxation of the water protons [6,7]. There are no reports on Cr-CEST imaging using an inverse Z-spectrum analysis of the testis.

We aimed to investigate the usefulness of inverse Z-spectrum analysis in Cr-CEST to evaluate mice with cisplatin-induced testicular dysfunction, by comparing the conventional analysis metric *MTR_conv_* and a novel metric *AREX,* using preclinical 7T-magnetic resonance imaging (MRI).

## 2. Materials and Methods

### 2.1. Animal Preparation

All animal procedures were approved by the research ethics committee of our university. All experimental procedures involving animals and their care were performed in accordance with the Osaka University Guidelines for Animal Experimentation. We purchased C57BL/6 male mice from SLC Japan (Shizuoka, Japan). The 16 mice were divided into 3 groups (aged 7–8 weeks). First, we performed an experiment using control mice (6 mice, 12 testes, aged 9–10 weeks) to demonstrate the in vivo application of inverse Z-spectrum analysis. To assess the impaired testes, cisplatin was administered to the mice. Cisplatin is an anticancer drug used in chemotherapy and causes testicular dysfunction [21,22,23,24]. To observe the dose dependence effect of cisplatin on testicular function, we prepared two groups using different doses of cisplatin (Wako, Osaka, Japan), as follows: a low-dose group (7.5 mg/kg, 6 mice, 12 testes, aged 9–10 weeks) and a high-dose group (15 mg/kg, 6 mice, 12 testes, aged 9–10 weeks). Cisplatin was administered via an intraperitoneal injection. We used all mice after a cisplatin intraperitoneal injection. We performed CEST imaging 18 days following the cisplatin injection. During all animal experiments, the mice were placed under general anesthesia with isoflurane (3.0% for induction and 1.5% for maintenance). Their body temperature was maintained at 36.5 °C with regulated water flow, and their respiration was continuously monitored using a physiological monitoring system (SA Instruments, Inc., Stony Brook, NY, USA).

### 2.2. MRI Experiments

All MRI experiments were performed on a horizontal 7T-MRI unit (PharmaScan 70/16 US, Bruker BioSpin, Ettlingen, Germany, 16 cm horizontal bore magnet, 9 cm inner diameter shielded gradient), equipped with a volume coil with a 30 mm inner diameter. We acquired CEST data using MT rapid acquisition with a refocused echoes (RARE) sequence. The sequence parameters were as follows: repetition time (TR) = 2300 ms; echo time (TE) = 33 ms; field of view (FOV) = 25.6 × 25.6 mm^2^; slice thickness = 1 mm; matrix size = 128 × 128; number of averages = 1; and in-plane resolution = 200 × 200 µm^2^. The MT pulse parameters were as follows: shape of continuous-wave saturation pulse = block pulse; length = 100 ms; number of pulses = 20; interpulse delay = 0.01 ms; bandwidth = 12.8 Hz; B_1_ amplitude = 1.2 µT; and flip angle = 1840°.

We obtained the Z-spectrum from CEST images with varying saturation frequencies, ranging from −4.8 ppm to +4.8 ppm (0.3 ppm per step; 33 images), and 1 S_0_ image (without an MT pulse). Point-by-point B_0_ correction was performed by water saturation shift referencing (WASSR) [25]. To obtain the WASSR data, the B_1_ amplitude was set to 0.3 µT and saturation offsets were set from −1.0 ppm to +1.0 ppm (0.1 ppm per step; 21 images). For the inverse Z-spectrum analysis, we obtained T_1_ map images. These maps were acquired using the T_1_ map RARE sequence. The sequence parameters were as follows: TE = 16 ms; TR = 5500, 3000, 1500, 800, 400, and 200 ms; FOV = 25.6 × 25.6 mm^2^; slice thickness = 1 mm; matrix size = 128 × 128; number of averages = 1; in-plane resolution = 200 × 200 µm^2^; and RARE factor = 4. The total acquisition time per animal was approximately 60 min.

### 2.3. Data Processing

All processing and data analyses of the CEST imaging were performed using in-house scripts written in MATLAB (2021a, MathWorks, Inc., Natick, MA, USA). MTR values were obtained using ImageJ (National Institutes of Health, Bethesda, MD, USA) from the CEST images created by the MATLAB analysis. In animal experiments, the oval regions of interest (ROIs) were attributed to the entire left and right testes to measure their signal value. In the experiment using control mice, we set the ROIs in the left and right testicular epithelium to observe the change in contrast before and after the inverse Z-spectrum analysis. We calculated and compared the MTR values and differences in MTR asymmetry between the testes and testicular epithelium (ΔMTR asymmetry). ΔMTR asymmetry is defined as follows:

Contrast of MTR asymmetry (%) = MTR (testis) − MTR (testicular epithelium).

In this study, the conventional MTR asymmetry was described as *MTR_conv_*. *MTR_conv_* and *AREX* were calculated as previously described [6,26]. The most common CEST quantification method, Z-spectrum asymmetry analysis, uses the subtraction of the Z values between Z_lab_ (the label scan at the resonance frequency offset of the target metabolite) and Z_ref_ (the reference scan at the frequency located symmetrically to 0 ppm).

*MTRconv* = Z_ref_ − Z_lab_

where *MTR_rex_* is the spillover- and MT-corrected index derived from the *AREX* calculation process.

(1) $${\mathrm{MTR}}_{\mathrm{rex}}=\frac{1}{Z_{\mathrm{lab}}}-\frac{1}{Z_{\mathrm{ref}}}$$

In addition, considering the T_1_ relaxation compensation, the AREX is calculated as follows:

(2) $$\mathrm{AREX}=(\frac{1}{Z_{\mathrm{lab}}}-\frac{1}{Z_{\mathrm{ref}}})/T_{1}=\frac{MTR_{rex}}{T_{1}}$$

### 2.4. Histopathology

Following MRI, we sacrificed the control and cisplatin-treated mice. The testes were collected and fixed in 4% phosphate-buffered formaldehyde (control: three mice, six testes; low-dose: three mice, six testes; and high-dose: three mice, six testes). Testis specimens were embedded in paraffin, sectioned at 5 µm, and stained with hematoxylin and eosin. The tissues were dehydrated with 95% ethanol twice for 30 min, and then soaked in xylene for 1 hour at 60–70 °C, followed by paraffin for 12 h. For the mouse testes, we used 0.5 mL of 95% ethanol in dehydration. We examined the testicular tissues using a light microscope (Keyence, Osaka, Japan). For the histological evaluation of spermatogenesis, we evaluated at least 20 seminiferous tubules per testis to calculate the Johnsen score [27], a 10-point evaluation method for quantifying spermatogenesis according to the profile of cells encountered along the seminiferous tubules. A Johnsen score of 10 indicates complete spermatogenesis with several spermatozoa, whereas a score of 1 indicates no cells in the spermatogonia. The Johnsen score was evaluated by one urologist with >5 years of clinical experience.

### 2.5. Statistical Analysis

The MTR asymmetry values are presented as mean values ± standard deviation (SD). We performed a Wilcoxon’s test to compare the differences between the two groups (*MTR_conv_* vs. *AREX*) using Prism 8 (version 8, GraphPad Software, San Diego, CA, USA). The differences between the three groups (control, low-dose and high-dose) were analyzed using the Kruskal–Wallis test. Differences were considered statistically significant at *p*-value < 0.05. The notations for significant differences in the graphs of results are defined as follows: * *p* < 0.05 and ** *p* < 0.01.

## 3. Results

### 3.1. In Vivo Imaging

#### 3.1.1. Application of Mouse Testes

**Figure 1:**
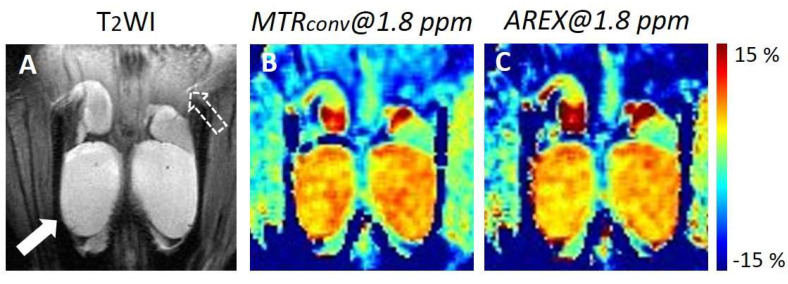
Representative in vivo Cr-CEST images of control mouse testes. (**A**) Anatomical T_2_WI image. The white arrow shows the testis. The white dotted arrow shows the testicular epithelium. (**B**) MTR asymmetry maps at 1.8 ppm of *MTR_conv_*. (**C**) MTR asymmetry maps at 1.8 ppm of *AREX*. MTR, magnetization transfer ratio; *MTR_conv_*, conventional analysis metric magnetization transfer ratio; *AREX*, apparent exchange-dependent relaxation; and Cr-CEST, creatine chemical exchange saturation transfer.

**Figure 2:**
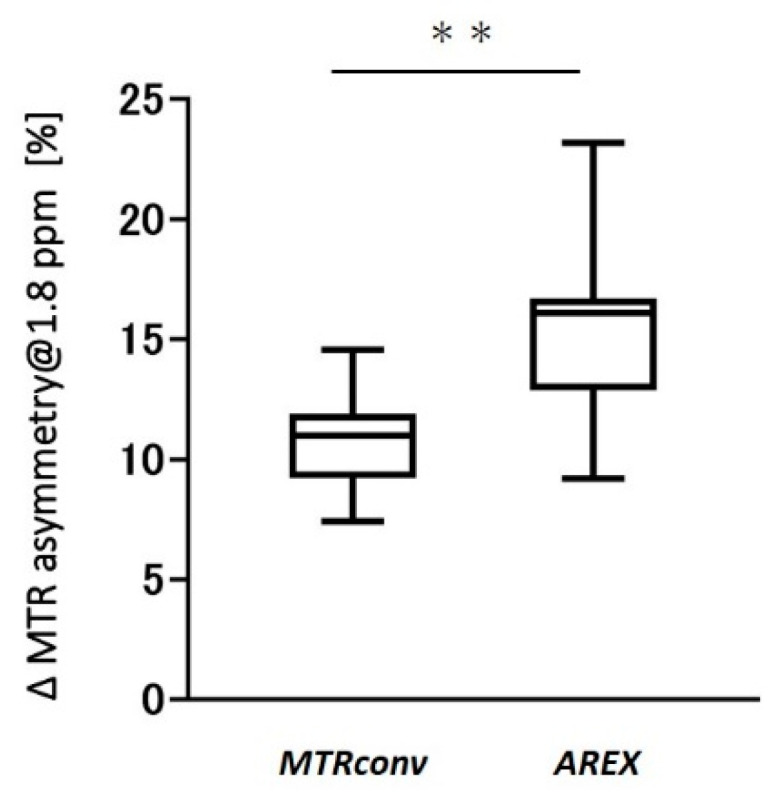
Difference in MTR asymmetry values between the testis and testicular epithelium at 1.8 ppm of *MTR_conv_* and *AREX.* ** *p* < 0.001. MTR, magnetization transfer ratio; *MTR_conv_*, conventional analysis metric magnetization transfer ratio; and *AREX*, apparent exchange-dependent relaxation.

Figure 1 depicts the representative in vivo Cr-CEST images of the mouse testes. In the MTR asymmetry map at 1.8 ppm, the testes displayed a higher MTR value than other tissues (Figure 1B,C). There was no visible difference between the MTR value of *MTR_conv_* and that of *AREX* in the testes. However, the MTR value of *AREX* was lower than that of *MTR_conv_* in the testicular epithelium. Figure 2 depicts the difference in the MTR asymmetry value at 1.8 ppm between the testis and testicular epithelium. The ΔMTR asymmetry of *AREX* was significantly larger than *MTR_conv_* (*MTR_conv_*: 11.0 ± 2.0 vs. *AREX*: 15.6 ± 3.5; *p* < 0.001).

#### 3.1.2. Cisplatin-Treated Model Observations

**Figure 3:**
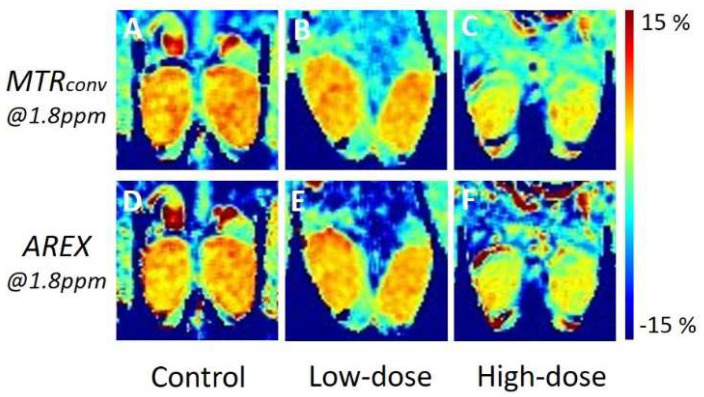
Representative in vivo Cr-CEST images of control and cisplatin-treated mice. The upper row displays the MTR asymmetry map of *MTR_conv_* at 1.8 ppm, whereas the bottom row displays the MTR asymmetry map of *AREX*. The left column represents the control (**A**,**D**), the central column represents the low dose (**B**,**E**), and the right column represents the high dose (**C**,**F**). MTR, magnetization transfer ratio; *MTR_conv_*, conventional analysis metric magnetization transfer ratio; *AREX*, apparent exchange-dependent relaxation; and Cr-CEST, creatine chemical exchange saturation transfer.

Figure 3 depicts the representative in vivo Cr-CEST images of the control mice and low- and high-dose cisplatin-treated mice. The MTR value of the cisplatin-treated mouse testes was lower than that of the control in both *MTR_conv_* and *AREX* (Figure 3A–C and Figure 3D–F). The high-dose model showed a larger reduction than the low-dose model. There was no visible difference between *MTR_conv_* and *AREX*.

**Figure 4:**
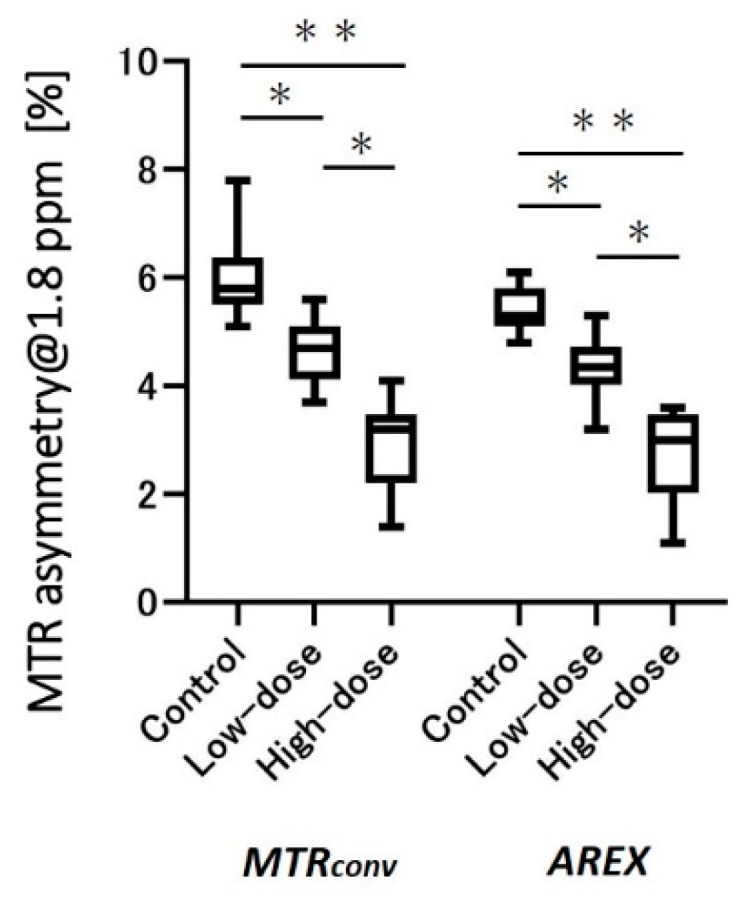
MTR asymmetry graphs at 1.8 ppm in the testes of control, low-, and high-dose cisplatin-treated models. * *p* < 0.05, ** *p* < 0.001. MTR, magnetization transfer ratio; *MTR_conv_*, conventional analysis metric magnetization transfer ratio; and *AREX*, apparent exchange-dependent relaxation.

Figure 4 contains graphs of MTR asymmetry at 1.8 ppm and the results of the statistical analysis. The low- and high-dose models revealed a more significant decrease than the control in both *MTR_conv_* and *AREX*. Furthermore, the high-dose group was significantly lower than the low-dose group (*MTR_conv_*, control: 6.0 ± 0.7 vs. low-dose: 4.6 ± 0.6, *p* < 0.05; control: 6.0 ± 0.7 vs. high-dose: 3.0 ± 0.8, *p* < 0.001; low-dose: 4.6 ± 0.6 vs. high-dose: 3.0 ± 0.8, *p* < 0.05. *AREX*, control: 5.4 ± 0.4 vs. low dose: 4.3 ± 0.5, *p* < 0.05; control: 5.4 ± 0.4 vs. high dose: 2.8 ± 0.9, *p* < 0.001; low-dose: 4.3 ± 0.5 vs. high-dose: 2.8 ± 0.9, *p* < 0.05). The low-dose model revealed a 23% and 21% decrease in the MTR value for *MTR_conv_* and *AREX*, respectively, compared with the control, whereas the high-dose model revealed a 50% and 48% decrease, respectively.

### 3.2. Histological Changes in Cisplatin-Treated Model

**Figure 5:**
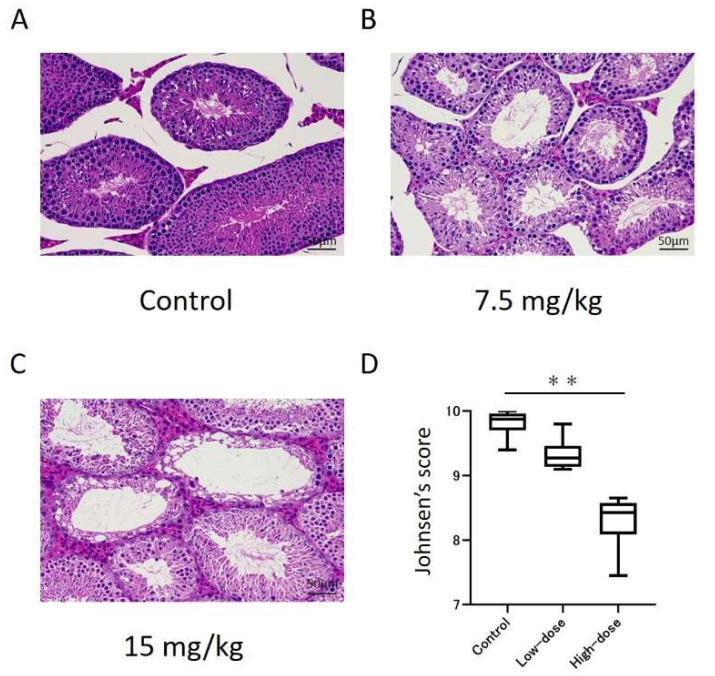
Histopathological changes in the testes of the control and cisplatin-treated mice. (**A**–**C**) Images of hematoxylin and eosin staining of the seminiferous tubules. (**D**) Graphical representation of the Johnsen score. ** *p* < 0.001.

Figure 4 represents the histopathological analysis of the testes in the control and cisplatin-treated mice. We observed spermatogenesis dysfunction in both the low- and high-dose cisplatin-treated models (Figure 5A–C). The Johnsen score for the high-dose group was significantly lower than that for the control (Figure 5D; control: 9.8 ± 0.2 vs. high-dose: 8.3 ± 0.4, *p* < 0.001), whereas there was no significant difference in the low-dose group (low-dose: 9.3 ± 0.2).

## 4. Discussion

This is the first study to report the efficacy of Cr-CEST imaging using inverse Z-spectrum analysis for assessing the dose dependence of anticancer drugs on testicular function. The application of in vivo imaging of mouse testes improved the specificity of the CEST effect and image contrast between the testis and testicular epithelium. The cisplatin-treated model displayed impaired testicular function, and Cr-CEST revealed decreased Cr levels in the testes. The decrease in Cr levels in the testes showed the dose dependence of anticancer drugs. The difference between the control and cisplatin-treated models was similar in the *MTR_conv_* and *AREX* groups. Cr-CEST imaging of the testis with inverse Z-spectrum analysis may help to assess the effects of anticancer drugs on testicular function.

### 4.1. Application of Mice Testes

The testis is an organ rich in Cr, particularly in the Sertoli cells and germ cells [28]. Takahashi et al. reported that the MTR value of the testis is five times higher than that of the skeletal muscle in Cr-CEST [19]. Despite the unclear role of Cr in the testis, infertile testes [29], ischemic testes [19], and X-ray-exposed testes [20] reveal low Cr levels. Moreover, Cr is a supposedly useful biomarker of testicular function. In this study, the testes displayed strong Cr-CEST signals. Moreover, Cr-CEST imaging using inverse Z-spectrum analysis revealed a significant increase in contrast between the testis and testicular epithelium in *AREX*, thus indicating increased specificity of the CEST effect. Cr-CEST imaging using inverse Z-spectrum analysis was also useful for detecting Cr in the testes.

### 4.2. Anticancer Drug Administration Model Observations

We not only examined changes in the MTR value between *MTR_conv_* and *AREX* in the control mice, but also the usefulness of inverse Z-spectrum analysis in a model of induced testicular dysfunction. We compared the difference in MTR values between the control and model mice with cisplatin-induced impaired testicular function, before and after inverse Z-spectrum analysis. By comparing the CEST images at 1.8 ppm obtained from the control, low- (7.5 mg/kg) and high-dose (15 mg/kg) cisplatin-treated models (Figure 3), we found that there was a visible difference between the three groups in both *MTR_conv_* and *AREX*. Similarly, Figure 4 revealed that in both *MTR_conv_* and *AREX*, the MTR value of the cisplatin-treated groups was significantly lower than that of the controls, and there was also a significant difference between the low- and high-dose models. This revealed that the impairment of testicular function with cisplatin administration is dose dependent. There was no major difference in the MTR values of the testes between *MTR_conv_* and *AREX*, and both showed similar trends. Although the contrast in the images was increased in *AREX*, the MTR values of the testes did not change substantially before and after inverse Z-spectrum analysis, indicating that the MTR values of the testes were not greatly affected by inverse Z-spectrum analysis.

In this study, which is focused on testis pathology, we observed spermatogenesis in both the control and cisplatin-treated models, but the testis of the cisplatin-treated mice was damaged and its cell structure was disorganized. The degree of impairment was more severe in the high-dose model compared to the low-dose model, and the Johnsen score showed similar results. Previous studies administered cisplatin at 2, 4, 6, 8, and 10 mg/kg, and the Johnsen scores were lower with a dose dependence [30]. Our result was consistent with this. However, in the comparison of the MTR values, the low-dose model showed a significant decrease compared to the control, and there was also a significant difference between the low- and high-dose models, whereas the Johnsen score only showed a significant difference in the high-dose model. Therefore, Cr-CEST imaging may be more sensitive to testicular dysfunction caused by anticancer drug administration than histopathology by biopsy.

In this study, the CEST images revealed that the administration of anticancer drugs impaired testicular function and decreased Cr levels. In addition, the degree of testicular damage was dose dependent for cisplatin, and the MTR values of the testes in Cr-CEST imaging were found to reflect this. This trend remained after inverse Z-spectrum analysis. Therefore, Cr-CEST imaging using inverse Z-spectral analysis may be applied to the evaluation of testicular Cr levels in pathological models.

### 4.3. Limitation

This study had one limitation: we only performed B_0_ correction using the WASSR method to acquire the CEST imaging data, and performed no correction for B_1_ inhomogeneity. The CEST effect depends on the B_1_ amplitude and is maximal at a certain amplitude [11]. Thus, B_1_ inhomogeneity not only affects the signal-to-noise ratio of the CEST signal, but also the image contrast [26]. *MTR_conv_* is not easily affected by B_1_ inhomogeneity, and the changes in image contrast with and without B_1_ correction are small. However, *AREX* is substantially dependent on B_1_ inhomogeneity. Without B_1_ correction, the entire FOV may have an inhomogeneous contrast [7]. In particular, we performed the present experiment using ultra-high field 7T-MRI with large B_1_ inhomogeneity [26]. This necessitates B_1_ correction or RF shimming for CEST imaging using inverse Z-spectral analysis in ultra-high field MRI.

## 5. Conclusions

We applied inverse Z-spectrum analysis to in vivo Cr-CEST imaging using a preclinical 7T-MRI, which improved the specificity of the CEST effect. The MTR values of Cr-CEST reflected the cisplatin dose dependence of testicular dysfunction.

## Data Availability

The data presented in this study are available on request from the corresponding author.
